# Non-canonical Roles of Complement in the CNS: From Synaptic Organizer to Presynaptic Modulator of Glutamate Transmission

**DOI:** 10.2174/011570159X327960240823065729

**Published:** 2025-01-14

**Authors:** Anna Pittaluga, Veronica Torre, Guendalina Olivero, Nicole Rosenwasser, Alice Taddeucci

**Affiliations:** 1 Department of Pharmacy, DIFAR, Pharmacology and Toxicology Section, Centre of Excellence for Biomedical Research, 3Rs Center, University of Genoa, viale Cembrano 4, Genoa, 16148, Italy;; 2 IRCCS Ospedale Policlinico San Martino, Genova, 16145, Italy;; 3 Department of Pharmacy, DIFAR, Pharmacology and Toxicology Section, University of Genoa, Viale Cembrano 4, Genoa, 16148, Italy

**Keywords:** Complement, C1q, C3, presynaptic, glutamate, glutamate transporter, glutamate receptor, antibody-antigen complex

## Abstract

The central nervous system (CNS) is not an immune-privileged compartment, but it is intimately intertwined with the immune system. Among the components shared by the two compartments is the complement, a main constituent of innate immunity, which is also produced centrally and controls the development and organization of synaptic connections. Complement is considered a doubled-faced system that, besides controlling the physiological development of the central network, also subserves synaptic engulfment pivotal to the progression of neurodegenerative diseases. Quite interestingly, besides these “*canonical”* roles, evidence in the last two decades highlighted other “*non-canonical*” role(s), thereby complementing modulates chemical transmission at central synapsis. It emerged that glutamate is the preferential target of these “*non-canonical*” complement-induced effects, which include i) the control of the release of glutamate from neurons and astrocytes and ii) the control of the number and the functions of central glutamatergic receptor subtypes (*i.e*., the NMDA receptors, the AMPA/kainate receptors, and the metabotropic glutamate receptors) in plasma membranes. This review summarizes some of the available results supporting the role of complement as a “*modulator”* of central glutamate transmission, paying particular attention to those events that occur presynaptically. Taking into consideration the enormous progress in complement pharmacology and the increasing number of therapeutics in clinical trials, deepening our knowledge of these” *non-canonical*” role(s) could pave the road to new therapeutic approaches for the management of central neurological diseases.

## 1 INTRODUCTION

### 1.1 Complement as a Synaptic Organizer

The term “*synaptic organizer*” relates to those molecules that regulate the development, organization, and removal of synapses in the central nervous system (CNS). It includes “*presynaptic organizers*” that control the specialization of the presynaptic boutons and “*postsynaptic organizers*” that preferentially recruit receptors/proteins, strengthening or destabilizing synaptic contacts [1-5].

Complement recently entered this class of compounds, based on the findings that its components and the related receptors i) are physiologically expressed in several central regions, ii) dictate the development and functions of the synaptic connections throughout the individual’s lifespan, and iii) modulate the efficiency of excitatory transmission [6-8].

Complement consists of over thirty soluble or membrane-associated proteins and the related cognate receptors. The soluble complement proteins circulate in the inactive form but can be cleaved to produce smaller active fragments through three major pathways (*i.e*., the lectin pathway, the classical pathway, and the alternative pathway). Specifically, C1q preferentially initiates the classical pathway, while the C3 factor triggers the alternative one [9-12]. C3 also activates the C5 convertase, leading to the production of the C5a component, which participates in the assembly of the pore-forming membrane attack complex (MAC) involved in cell lysis (*i.e*., the lectin pathway [13]).

In the CNS, the complement has been described as a double-edged sword able to control the physiological development of the brain but, in the meantime, to account for detrimental events that typify the progression of central neurodegenerative/neuropsychiatric diseases [9, 14, 15]. Improving the knowledge of the central roles of this system would unveil new aspects of the immune-mediated modulation of the brain and suggest new approaches for therapies to protect synaptic networks throughout the lifespan.

A large part of the literature concerning the role of complement as a “synaptic organizer” focusses on glutamatergic synapsis. Glutamate is the major excitatory transmitter in the brain, which mediates the chemical transmission at neuronal synapses. It acts as an excitatory relay between the presynaptic nerve terminals and the postsynaptic counterpart. Synapsis is composed of both presynaptic and postsynaptic components, which, by facing one another, define the synaptic active zone. The presynaptic component synthetizes and stores glutamate in vesicles bearing the vesicular glutamate transporters (VGluTs) and releases it in response to depolarizing stimuli. Presynaptic terminals also possess glutamate transporters that take up glutamate from the synaptic cleft, controlling its bioavailability and maintaining it around low physiological levels. Once released in the synaptic cleft, glutamate activates both ionotropic and metabotropic glutamate receptors located either postsynaptically (to assure the progression of the excitatory signal) or presynaptically (to finely tune the intensity and the duration of presynaptic glutamatergic overflow) [16].

The dynamic of synaptic communication at glutamatergic synapsis, however, is also influenced by astrocytes, which embrace neuronal synapsis to support and control their functions in a tripartite structure. The term tripartite synapse was coined by Araque and colleagues in 1999 [17] to emphasize the direct participation of astroglia in synaptic signalling, along with presynaptic nerve endings and postsynaptic neurons. Specifically, astrocytes release and take up glutamate, and the efficiency of these two events indirectly influence the bioavailability of the aminoacid in the synaptic cleft and, consequently, the efficiency and the strength of the chemical communication at the tripartite synapsis.

Neuronal synaptic connections might involve the presynaptic components of short axonal processes (originating from nearby neuronal cells) or long-distance axonal processes (*i.e*., from neurons located far from the synaptic contact), which assure either a short or a long-distance propagation of the chemical stimulus to optimize neuronal development and brain communication. Astrocyte glutamatergic signal, on the contrary, originates from astrocytic specializations within the tripartite synapsis and influences the strength of synaptic connection locally. The equilibrium between the neuronal and the astrocytic glutamatergic signals determines the efficiency of the synaptic communication and, if impaired, becomes pathogenic. Several are the reviews that have been dedicated during the last 30 years to these aspects, and that can be consulted for more information [18-20].

This review is dedicated to summarizing the available results concerning some “*non-canonical*” roles of complement as a *modulator* of glutamatergic signal in the CNS, specifically, those events that preferentially occur presynaptically and that could be functional to the excitatory transmission in physiological and pathological conditions. The presynaptic role of complement as a modulator of excitatory transmission indicates this immune complex as one of the most attractive targets for new therapies for the management of neuropsychiatric and neurodegenerative diseases [21, 22].

## 2 PRESYNAPTIC COMPLEMENT PROTEINS IN THE CENTRAL NERVOUS SYSTEM

The existence of complement proteins and cognate receptors in mammals was first proposed based on results from *in situ* hybridization in autoptic human brain tissues and rodent CNS samples [3, 23-25]. Microglia, astrocytes, oligodendrocytes, and neurons were found to be competent for complement proteins (particularly the C1q, the C3, and the C5a proteins) and to release them in a fashion strictly dependent on the concomitant physio-pathological conditions. These cells were also reported to be endowed with the cognate receptors to transduce the signal elicited by the complement proteins (*i.e*., C3aR, C5aR, and others) [3, 8, 11].

As far as the glia is concerned, the accumulation of C1q/C3 usually was associated with the pathological activation of these cells (*i.e*., C1q and C3 are proposed as selective markers of reactive type A1 astrocytes) [26-28] and with the mechanisms of synaptic stripping and pruning [3, 4, 12, 28, 29].

Besides glial cells, C1q, C3, and C5 are also expressed in neurons, specifically in the synaptic compartments [3, 8, 12], where they accumulate upon exposure to developmental cues during the entire life [30] or in the course of neurodegenerative (Parkinson’s or Alzheimer’s disease) and autoimmune (multiple sclerosis, optic neuritis) diseases [12, 29, 31]. The following sections are dedicated to revising part of the available literature supporting the presynaptic distribution of the C1q, the C3, and the C5a components in the CNS of mammals.

### 2.1 Presynaptic C1q in the CNS

C1q is expressed in neurons at all developmental stages, in proximity to synaptic terminals (in neuronal processes immune-positive for synaptophysin, SYN) [31-33]. Here, the level of C1q increases during aging, consistent with its role in the reorganization and simplification of the neuronal contacts and with the concomitant cognitive decline [30, 34, 35]. C1q localizes both in synaptic puncta and in axons, and its expression is controlled by other synaptic organizers, including some cytokines (*i.e*., IL-1a, IL-1b, TNF-a) [3].

In 2007, Stevens and colleagues demonstrated that, in mice, C1q is upregulated in neurons purified from the developing eye (*i.e*., the retinal ganglion cells, RGCs), starting from postnatal day 5 [36]. Specifically, the C1q immunopositivity was detected in retina synaptic puncta between postnatal P4 and P40, and it was colocalized with presynaptic (synaptic vesicle glycoprotein 2, SV2) and postsynaptic (post-synaptic density protein 95, PSD95) markers, consistent with its presence at both sides of synaptic contacts. Interestingly, a preferential presynaptic colocalization with SV2 was also detected in small synaptic puncta that lacked a postsynaptic partner, which the authors proposed might represent immature synapses or synapses in the process of elimination. The triggering signal producing the C1q in RGCs was proposed to originate from surrounding astrocytes. This cascade of events also leads to the accumulation of C3 [9] and consequent recruiting of C3 receptor-bearing cells (microglia cells and astrocytes), promoting synaptic engulfment. Interestingly, the authors demonstrated that this developmentally regulated pathway becomes aberrant in pathological conditions associated with glaucoma.

Stephan and colleagues also proposed a presynaptic localization of C1q [30]. The authors highlighted a significant C1q immunoreactivity to and near the surface of presynaptic elements. Unfortunately, the analysis did not allow for the tri-dimensional reconstruction and quantification of synaptic vs. non-synaptic distribution of these components. C1q immunostaining was detected near structures bearing anti-SYN immunoreactivity, which could be identified as presynaptic terminals.

Matsuda and colleagues also proposed the role of C1q as a preferential presynaptic organizer [37-40]. By investigating the mechanisms underlying the synaptic organization in the cerebellum, the authors demonstrated that C1q proteins, particularly the Cbln1, accumulate at the presynaptic contacts of the parallel fibers. Here, Cbln1 controls the correct assembly of the parallel fiber-Purkinje cell synapses through a mechanism involving the presynaptic protein neurexin. The Cq1-related protein released from the presynaptic component of the chemical synapsis was also proposed to determine the correct positioning of kainate receptors at the postsynaptic counterpart, controlling the strength of the synaptic connection. Notably, this role is not confined to the cerebellum, but it also occurs in other regions, including the hippocampus, where the mRNA encoding for the C1q-like subfamily is highly expressed in the dentate gyrus granule cells that send mossy fiber processes onto pyramidal cells in the CA1 region [1, 41]. C1q and C1q-like proteins emerged with a prominent role in the organization of the pre-and post-synaptic components of active synapsis, specifically in the maturation of the presynaptic terminals. Furthermore, the studies described molecular events and ancillary proteins that could drive the C1q-mediated mobilization of selected glutamate receptor subtype (the kainate, the AMPA, and the GluD2 -containing receptors, see [1] and section 3.3 of the present review).

In 2015, Michailidou and colleagues performed double immunofluorescent staining for the presynaptic marker SYN and for C1q in autoptic hippocampi of patients suffering from multiple sclerosis (MS) and found a huge colocalization of C1q and SYN at the hippocampal CA3 and CA1 areas, irrespective of the presence of de-myelinated regions. The authors also highlighted the lack of evident immunoglobulin depositions in the C1q/C3 immuno-positive regions but the presence of clear staining for C5b9, which, in their opinion, supports the role of complement receptors in synaptic internalization/pruning [31].

The presence of C1q at the presynaptic compartment of central synapsis was definitively proven in 2018 [33]. By using the Fluorescence Activated Cell Sorting (FACS) technique combined with proteomics analysis, the authors unambiguously proved the presence of C1q in presynaptic nerve endings as well as in synaptosomes from the cortex of mice. The authors also investigated the distribution of the C1q protein in cortical synaptosomes and found that it preferentially accumulates in the presynaptic components of the synaptic active zone. The C1q expression increased during aging and correlated with apoptotic markers and with the phosphatidylserine protein, which was proposed as a potential binding partner of C1q. These observations were confirmed later [42]. C1q was reported to colocalize presynaptically also with neuronal pentraxin (NPx), and their colocalization was related to the efficiency of synaptic pruning [43]. The authors also demonstrated a significant synaptic colocalization of C4, which they hypothesized might have a role in the C1q-NPx synaptic loss. The presynaptic localization of the C1q proteins also was confirmed by Kaway and colleagues [44]. It was found that C1q is expressed in the circumventricular organs, where its density increases following a single administration of lipopolysaccharide. Lastly, we recently demonstrated the presence of clear C1q immunoreactivity in isolated cortical nerve endings (synaptosomes) from healthy mice and found that it is overexpressed in pathological conditions associated with autoimmune responses [45].

### 2.2 Presynaptic C3 in the CNS

Complement C3 is the point of convergence of the three major cascade pathways (the classical, the alternative, and the lectin ones). Its functions are mainly mediated by the opsonin C3b/iC3b and the anaphylatoxin C3a. The expression of functional C3 was identified in “*in vitro*” human neuronal cells [46] and in human neurons in postmortem brain tissue, where the mRNA coding for C3 was detected by *in situ* hybridization [31]. In 2015, Michalaidou and colleagues [31] highlighted the presence of C3 immunopositivity in neurons and in synaptic terminals, where the C3d fragment (which represents the end-product of the C3 protein cleavage) was maximally expressed. Interestingly, the immunopositivity increased during MS progression. They also found a huge increase in the C3 alpha chain, which, they proposed, could be predictive of the activation of C3-mediated opsonization of synaptic processes. Overall, their results support the involvement of C3 in the pathological tagging of presynaptic nerve terminals. Specifically, the authors concluded that, in concert with C1, C3 could promote the phagocytosis of presynaptic boutons by microglia recruited *via* C3R-dependent signals, irrespective of the level of demyelination and inflammation in the hippocampal regions. We recently confirmed the presence of C3 immunoreactivity in nerve endings isolated from the cortex of healthy mice. Again, we found a huge over-expression of this protein in cortical synaptosomes isolated from animals suffering from experimental autoimmune encephalomyelitis when compared to controls [47].

### 2.3 Presynaptic C5 in the CNS

The production/accumulation of complement C5 at synaptic connections has been inferred based on several studies, but further investigation is needed to support the hypothesis. C5a represents the final product of the cleavage of C1q and C3 fragments, and it is involved in the MAC-mediated lysis of cells. In 2009, Trent Woodruff and his group demonstrated that the G-protein coupled C5a receptor (*i.e*., the CD88) locates presynaptically on the axonal processes of granule cells (the mossy fibers) in the stratum lucidum of the hippocampal CA3 region [11]. The conclusion relied on the results from immunolabelling studies and electron microscopy analysis, which unveiled a diffuse colocalization of the CD88 protein with presynaptic markers, including SYN and synapsin-1 [48]. The presynaptic CD88, which is expected to mediate the C5a effects, was found to control calcium influx, as indeed also observed in hippocampal cultured neurons [48] or, more recently, in cortical neurons exposed to oxygen-glucose deprivation-reoxygenation [49]. Notably, the C5aR-mediated control of calcium dynamic was proposed to be relevant to central disorders. These authors also demonstrated that C5a increases calcium availability in hypothalamic neurons, controlling their secreting activity [50]. Besides these observations, the factor C5 was also reported to behave as a binding partner of oligomeric α-synuclein. A proteomic analysis unveiled that the complement system is differently expressed in two mouse models of Parkinson’s disease and accounts for α-synuclein dependent neuronal loss. Lastly, anti-C5 monoclonal antibody treatment (24 h after the disease induction) restored the strength in two-thirds of rats suffering from passive experimental Miastenia Gravis induced with acetylcholine receptor antibody McAb-3 [51-54]. In 2013, Gong and colleagues [55] demonstrated that C5a complement participates in maintaining hippocampal synaptic plasticity and efficient learning and memory in mice. They also provided evidence that, if overexpressed, C5a upregulates the AMPA receptors density and functions at active synapses by modulating the calcium calmodulin II (CaMKII)- cAMP Response Element-Binding Protein(CREB)-C pathway.

## 3 COMPLEMENT AT GLUTAMATERGIC SYNAPSES: FROM PRESYNAPTIC ORGANIZER TO PRESYNAPTIC MODULATOR OF GLUTAMATERGIC TRANSMISSION

Neurotransmitters act locally, supporting the “wiring transmission,” *i.e*., the intercellular communication which accounts for fast “point-to-point” communication at synaptic contacts, but also diffuses out of the synaptic cleft to reach receptors located on cells far from the site of release through the mechanism of the “volume diffusion” [56-61]. Glutamate supports these forms of synaptic communication: events that reduce or, more frequently, amplify its availability are pathogenetic in nature since they impair the chemical connection within the cellular network and support the progression of excitotoxicity.

Differently, neuromodulators preferentially modulate, rather than activate, chemical transmission by diffusing in the extracellular space through the mechanism of “volume diffusion,” also acting far from the site of release. Neuromodulators preferentially exert their effects by activating metabotropic receptors and/or by controlling enzymatic pathways [59, 62, 63]. Among all the candidates, complement recently emerged in the interest of scientists because of its efficiency in “tuning” chemical “wiring transmission,” specifically the excitatory one. As a matter of fact, the recent literature here suggests that the CNS complement can “modulate” glutamate transmission i) directly by controlling the release of the excitatory aminoacid at both neuronal processes and astrocytic specializations [47, 64] and ii) *indirectly* by influencing the number and the functions of glutamatergic receptor subtypes (*i.e*., the NMDA receptors, the AMPA/kainate receptors and the metabotropic receptors [38, 58, 65]. These are the observations that, in our opinion, support the hypothesis of a “non-canonical role” of complement as a modulator of excitatory transmission and that pose the basis for verifying the efficacy of complement ligands/modulators to manage the central glutamatergic transmission If confirmed, the hypothesis would open new approaches to pharmacological intervention for central diseases, potentially satisfying the so far unmet need of therapeutics for excitotoxic events, due to the scarce transability of glutamatergic ligands into clinic [21, 22].

Based on this assumption, the section is dedicated to revising the available literature concerning complement as a modulator of glutamate transmission. To this aim, we first discuss the data concerning the role of complement as a direct “modulator “of glutamate release, and then, we resume some findings unveiling mechanisms of autoactivation of complement that could lead to the local overexpression of complement components (*i.e*., the C1q and the C3) and the consequent amplification and prolongation of the complement-mediated control of glutamate overflow. Lastly, we will revise the available literature, which suggests that complement is a “meta modulator” of pre-and post-synaptic glutamate receptors in the CNS [66].

### 3.1 Complement as a Presynaptic Modulator of Glutamate Release

The role of complement in dictating the mechanism of vesicular exocytosis was indirectly inferred by Benoit and Tenner in 2011 [67]. The exposure of rat primary cultured neurons to C1q caused a significant upregulation of the gene encoding for syntaxin-3 (stx-3), which persisted over time. Quite interestingly, the C1q-induced overproduction of stx-3 was paralleled by an increased interaction of this protein with Synaptosomal-Associated Protein, 25kDa (SNAP25). The density of SNAP25 has conserved in C1q-treated cultured neurons with respect to untreated cells, nor was the gene upregulated, but the association between stx-3 and SNAP25 was implemented. The authors discussed the event as a C1q-induced neuroprotective effect, focusing on its potential beneficial impact on neuronal survival. The observation, however, also could be consistent with a positive impact of the C1q protein on the fusion events that dictate the efficiency of vesicular exocytosis and, therefore, the release of transmitters.

Soon later, in 2014, Perez-Alcazar and coworkers [64] used animals lacking the expression of the C3 component to investigate whether complement could have a role in the mechanism(s) subserving synaptic learning. In the hippocampus, the constitutive absence of C3 was found to affect the function of CA3-CA1 glutamatergic synapses. The authors demonstrated that the deletion of the C3 protein (C3 knock-out, ko, mice) impaired synapse elimination and altered synaptic communication. As far as the glutamate release probability was concerned, it emerged that CA3-CA1 synapses in the C3 ko mice exhibited a significantly higher paired-pulse ratio than wild type (wt) mice, consistent with the conclusion that the deletion of the C3 protein could have decreased release probability. Furthermore, dizocilpine (MK-801), an irreversible NMDA receptor channel blocker, showed a lower decay of the excitatory postsynaptic potential (EPSP) in C3 ko mice when compared to controls. Since the activation of postsynaptic NMDARs is strictly dependent on the release probability of glutamate from the presynaptic counterpart, it was proposed that, in the C3 ko mice, the glutamatergic input onto the postsynaptic component was less efficient than that in wt animals. The authors proposed that the altered postsynaptic responsiveness was not due to morphological or compensatory changes (*i.e*., the absence of C3 would be expected to reduce the synaptic pruning, preserving an excessive number of glutamatergic synapses) but rather to a reduced presynaptic input which might compensate for excessive glutamatergic synapses. On the whole, these findings indirectly pointed out the role of complement as a synaptic modulator of glutamatergic transmission in the hippocampus.

Almost concomitantly, we provided evidence that complement promotes the release of glutamate (measured as release of either endogenous glutamate and or preloaded [^3^H]D-aspartate, a non-metabolizable radioactive tracer which mimics glutamate in synaptic nerve endings) [58, 68, 69] from synaptosomes isolated from different central regions (the cortex, the cerebellum, the hippocampus, the spinal cord) of mice [70]. The complement-evoked releasing activity was not specie-specific since, besides mice, this event also was observed in rat cortical synaptosomes and even in nerve terminals isolated from human cortical specimens removed during neurosurgery to reach deep-located tumours. Inasmuch, complement did not release cytosolic proteins (*i.e*., the lactate dehydrogenase) concomitantly to glutamate, ruling out the possible involvement of unspecific lytic events (*i.e*., MAC-dependent events that disrupt the cell membrane favouring osmolysis) in the complement-evoked releasing activity.

From a mechanistic point of view, the complement-evoked glutamate outflow was almost totally prevented by the concomitant presence of the broad glutamate transporter blocker DL-threo-beta-Benzyloxyaspartate (dl-tBOA) or by reducing the [Na^+^]_out_ from 120 mM to 40 mM. Changes in the [Ca^2+^]_out/in_ were apparently less relevant to controlling the releasing activity. Specifically, neither the blockade of the L-type and of the N and P/Q-type channels nor buffering of the cytosolic calcium ions with BAPTA modified the complement-mediated control of glutamate release. We did not investigate the possible involvement of Ca^2+^ ions released from endoplasmic reticulum stores (which in other studies were proposed to underlie the complement-sensitive mobilization of the divalent cation in non-neuronal cells, *i.e*. neutrophil) [48, 71]. The possibility, however, seems unlike since, besides the lack of effect of BAPTA, pertussis toxin intoxication of synaptosomes (that would stop intraterminal GPCR-dependent mobilization of calcium ions from endoplasmic reticulum) did not modify the complement-induced effect, ruling out, in our opinion, the participation of calcium ions mobilized from internal stores in the releasing activity.

**Fig. (1):**
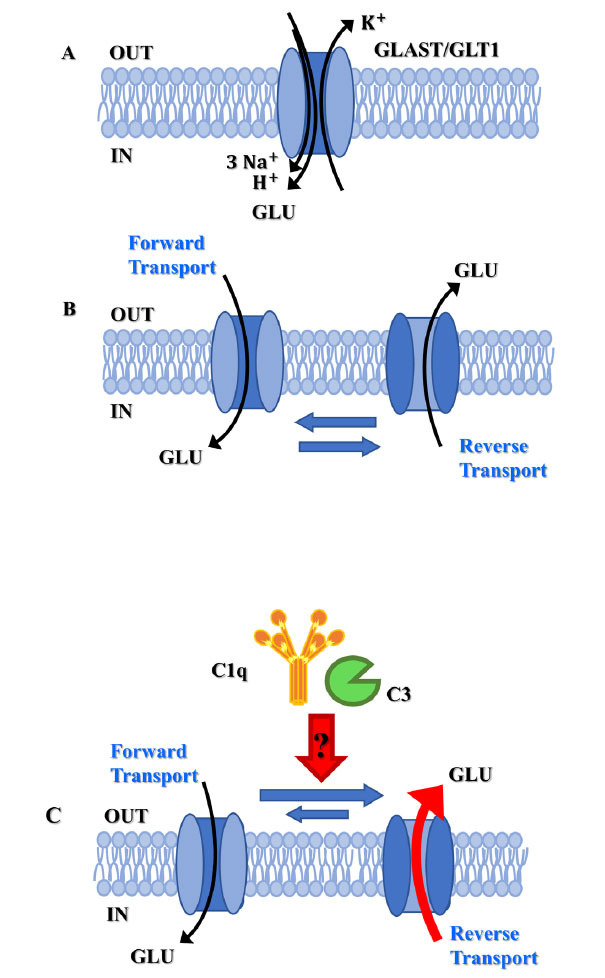
Complement releases glutamate through a mechanism involving the excitatory amino acid transporters GLAST/GLT1. (**A**) excitatory amino acid transporters GLAST/GLT1 (EAAT1/EAAT2 in humans) belong to the solute class 1 of transporters (SLC1). The forward transport of one molecule of glutamate is paralleled by the concomitant influx of 3 Na^+^ and 1 H^+^ cations and the concomitant exit of 1 K^+^. (**B**) GLAST/GLT1 cycles between two discrete conformational states, one allowing the forward transport of external glutamate and the other reverse transport of internal glutamate. (**C**) We propose that complement (C1q and C3) can accelerate the “alternating access mechanism,” promoting the transporter to work in the outward mode to release cytosolic glutamate.

Quite surprisingly, the releasing activity was restricted to glutamate since the spontaneous outflow of other transmitters (*i.e*., γ-amino-butyric acid, GABA, noradrenaline, serotonin, and acetylcholine) was unmodified in synaptosomes exposed to complement when compared to the untreated ones. The selectivity of complement towards glutamatergic particles appears consistent with the mechanism accounting for the complement-induced releasing activity that, based on the sensitivity to dl-tBOA, specifically engages the excitatory amino acid transporters glutamate aspartate transporter (GLAST)/glutamate transporter 1 (GLT-1) (excitatory amino acid transporter 1 (EAAT)1/EAAT2 in human, Figs. **1**, **1A**) which are expressed in glutamatergic terminals.

**Fig. (2):**
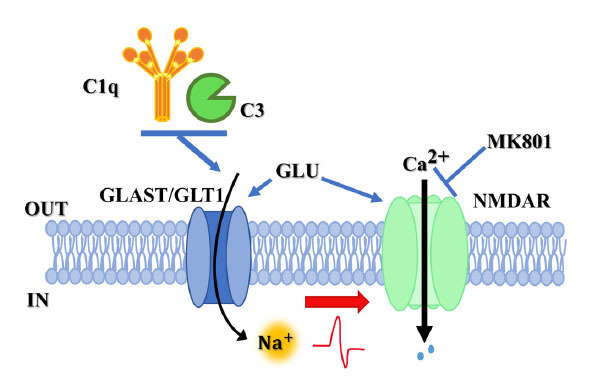
Complement favours the activation of colocalized presynaptic release-regulating NMDARs. Excitatory amino acid transporters GLAST/GLT1 are electrogenic in nature since the positive charge that flows out does not compensate for the four positive charges that flow in. We propose that complement (C1q and C3) can first promote the influx of cations that locally depolarize the membranes and favours the opening of the voltage-sensitive channel associated with the NMDAR located nearby the glutamate transporters, amplifying the releasing activity. When added concomitantly to complement, the NMDAR antagonist MK801 blocks the NMDARionic channels and prevents its participation in the complement-evoked releasing activity.

GLAST/GLT1 belongs to the solute class 1 of transporters (SLC1) and has different and specific cellular localization, which depends on the brain area [72, 73]. In general, astrocytes express both GLT-1 and GLAST, while axon terminals have a heterogeneous composition, the neocortical ones only expressing GLT-1 [74]. By removing excessive glutamate from the synaptic cleft, GLT1/GLAST transporters are neuroprotective and indirectly tune the [glutamate]_out_ at the glutamate receptor orthosteric binding site, maintaining their activation at the physiological level(s). GLT1/ GLAST transporters, however, can also work in the reverse mode, releasing glutamate through the mechanism of the “carrier-mediated release.” This event occurs at either nerve endings or astrocytic specializations [75] and depends on the mechanism(s) of homo-exchange, which can be ascribed to the so-called “alternating access” process [76]. Briefly, glutamate transporters can cycle through at least two discrete conformational states, one allowing the binding of external glutamate and the other of internal glutamate (Fig. **1B**). The first accounts for the uptake of glutamate, and the second one for glutamate outflow. The cellular events that favor the second state over the first one are far to be elucidated, but taking into consideration that the GLT1/GLAST-mediated uptake of one molecule of glutamate is paralleled by the concomitant influx of 3 Na^+^ and 1 H^+^ cations and the exit of 1 K^+.^, it might be hypothesized that the accumulation of Na^+^ ions at the inner side of plasma membrane (Fig. **1A**) (*i.e*. the positive charge that flows out does not compensate for the four positive charges that flow in) would cause a local mild depolarization and force the transporter to work in the reverse mode. Accordingly, reducing the net positive charges by lowering [Na^+^]_out_ prevents the EAAT-mediated processes [77] and largely hampers the complement-mediated releasing activity, as already introduced. The hypothesis that complement might cause a local depolarization pivotal to glutamate release is, in our opinion, indirectly supported by the finding that the complement-evoked glutamate outflow from hippocampal synaptosomes is reduced by the concomitant presence of dizocilpine, a selective NMDAR channel blocker [78]. As a matter of fact, the local depolarization elicited by the complement-evoked influx of Na^+^ ions could have favoured the opening of NMDAR-associated voltage-sensitive channels near the glutamate transporters, which might participate and amplify the complement-induced releasing activity and whose activation can be prevented by dizocilpine (Fig. **2**).

To conclude, although the role of GLAST/GLT1 in the complement-releasing activity remains so far largely unexplored, the huge sensitivity to dl-tBOA and the overt dependency on the [Na^+^]_out_ is consistent with a complement-mediated acceleration of the “alternating access mechanism,” that would promote the transporter to work in the outward mode to release cytosolic glutamate (Fig. **1C**). Consistent with this view, the uptake of glutamate was significantly reduced when synaptosomes were exposed to complement [79]. Whatever the mechanism(s) involved, the complement-induced releasing activity was nulled by removing the C1q or the C3 components, suggesting that this event preferentially depends on the classical pathway, which usually implies the interaction of complement with proteins stably anchored in plasma membranes and its autoactivation to accumulate locally C1q and then C3.

We recently extended the study to gliosomes [45], which originate from astrocytic specialization and are isolated concomitantly to synaptosomes. These particles permit the study of specific astrocytic events, including the release of glutamate. The releasing activity elicited by complement was largely reminiscent of that described in synaptosomes but occurred with higher efficiency.

Very recently, Lu and co-workers [80] demonstrated that C3 controls the presynaptic component of chemical synapsis. Their results, however, claim for an inhibitory, instead of a facilitatory, effect of C3 on glutamate transmission. The study was carried out with animals knocked out for miR-218-2, which is typified by an increased expression of C3 protein in the hippocampal neurons. These animals show a significant decrease in the miniature EPSPs frequency in the CA1 region, which would imply the reduction of the presynaptic vesicular release of glutamate. Since neither the postsynaptic expression of AMPARs nor the EPSP amplitude was modified, the authors proposed that the total synapse number was unmodified but that the readily releasable pool of synaptic vesicles was reduced when compared to the wild-type animals. These defects are well correlated with the reduced long-term potentiation detected in “*in vitro*” studies and the “*in vivo*” defective cognitive functions observed in these animals. Comparable effects were observed by infusing a purified C3 protein in the hippocampus of wild-type mice, while opposite effects were obtained by locally administering the C3 antagonist SB290157. Neither exogenous C3 nor SB290157 modified the morphology of the neurons, supporting the conclusion that both drugs preferentially controlled the mechanisms of glutamate transmission at chemical synapses rather than synaptic modeling.

A significant release of glutamate triggered by the C5a component of complement also was reported to occur in foetal cortical neurons [81]. The releasing activity was prevented by the concomitant presence of a C5a antagonist and is well correlated with the neurotoxic effect of the immune complex.

Another aspect discussed in the last few years that could support the hypothesis of complement as a controller of glutamate exocytosis is its modulating activity of presynaptic vGluTs expression, which indirectly would control the efficiency of glutamate release. In 2020, Gyorffy [42] and colleagues focused on septins, *i.e*., proteins that are engaged at the synaptic connections to modulate several functions. Septins belong to a highly conserved family of GTP-binding proteins which are divided into four subgroups: SEPT2 (which include the Septin 1, 2, 4, and 5 subtypes), SEPT3 (composed of the Septin 3, 9, 12), SEPT6 (including Septin 6, 8, 10,11, 14), and SEPT7 (Septin 7). Among these groups, the SEPT6s have a presynaptic distribution [82] and control vesicle trafficking by interacting with the vesicle-associated membrane protein 2 (VAMP2) and with syntaxin-1, promoting the formation of the SNARE complex and the docking of vesicles to the presynaptic membranes. Conversely, Septin 5 negatively regulates vesicular exocytosis at presynaptic terminals. In 2020, the group of Kardos (Giorffy *et al*. 2020) [42] identified increased levels of presynaptic septin 5 and 11 in C1q-tagged synapses in APP/PS1 mice, *i.e*., an amyloidogenic mouse model of Alzheimer’s disease. The authors correlated the overexpression of these proteins to a high compartmentalization of C1q at the presynaptic level and to an increased probability of developing presynaptic defects, including altered glutamate exocytosis. We propose that the septin-C1q cross-talk might provide a mechanism through which C1q over-tagging at presynaptic terminals could affect the efficiency of excitatory transmission in selected CNS regions.

To conclude, the data so far described support the hypothesis that complement can be viewed as a “direct modulator” of glutamate transmission in the CNS. Even more intriguingly, the results suggest that the “modulatory effect” is limited and restricted to the glutamatergic terminals since it cannot be observed when studying the release of other transmitters. The specificity of the releasing activity is impressive since, to the best of our knowledge, this is the first example of an endogenous compound that selectively modulates glutamatergic transmission. If confirmed, this specificity would account for the role of complement in dictating neuroplasticity and excitotoxicity in the CNS. Notably, some recent observations from our laboratory demonstrated that the complement-evoked releasing activity is not restricted to neurons but also occurs in astrocytes, where, however, it differs in terms of efficiency [45]. The physio-pathological implications of complement on chemical glutamatergic transmission and on the neuron-to-glia interaction would deserve future investigation for their relevance to the progression of central disorders [45].

### 3.2 Antigen-antibody Complexes Sensitize the Complement-induced Releasing Activity in Nerve Endings

The accumulation of the C1q and the C3 at active synapsis and, consequently, their impact on chemical communication does not occur autonomously but largely depends on auto-initiation mechanisms, which include the classical, the lectin, and the alternative pathways. Among them, the classical pathway is particularly attractive since it is activated by the interaction of C1q with triggering elements (*i.e*., the Fc domain of immunoglobulins or peptides) that typify neurodegenerative (*i.e*., the amyloid β1-42 oligomers in Alzheimer disease) [83] or autoimmune diseases (*i.e*., the anti-GluN or anti-GluA antibodies in autoimmune encephalitis) [84] and leads to a sustained overproduction of this component and downstream constituents (*i.e*., C3). Besides supporting widespread lytic events mediated by opsonins [85] and functional synaptic maladaptation, the over-accumulated C1q and C3 have been associated with altered neuronal responsiveness [86, 87] and, specifically, impaired glutamate transmission, as first hypothesized by Docherty and colleagues in 1983 [88].

As a matter of fact, the accumulation of the complement factors C1q and C3 elicited by amyloid β peptides was proven to mediate microglia-mediated synaptic engulfment and to alter synaptic long-term depression by modifying glutamate release and NMDA and metabotropic glutamate receptor type 5 (mGluR5)-mediated pathways [42, 86, 87]. Similarly, the overproduction of autoantibodies recognizing selected receptor subunits of glutamate receptors (*i.e*., antiGluN1 and GluN2 autoantibodies, and anti-GluA2 and 3 antibodies [84, 89] was proposed to activate complement-mediated neurodegenerative processes [90], but also to alter glutamate transmission by interfering with the targeted receptors or by affecting the probability of the complement-mediated releasing activity. To verify whether anti-AMPA or anti-NMDA antibodies might modify the complement-evoked release of glutamate, we incubated cortical and hippocampal synaptosomes (*i.e*. these terminals are endowed with presynaptic release-regulating AMPA and NMDA autoreceptors) with commercial anti-GluA and anti-GluN antibodies to promote the assembly of the antibody-antigen complex at the outer side of synaptosomal plasma membranes and to monitor the efficiency of the complement-evoked glutamate release from these terminals [91, 92].

**Fig. (3):**
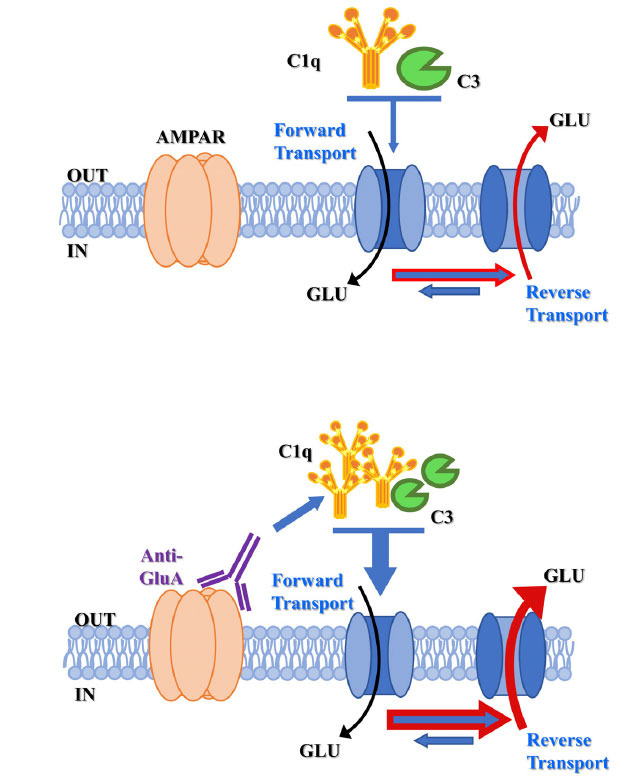
Anti-GluA antibodies amplify the complement-evoked releasing activity. The presence of anti-GluA/AMPA receptor complex in neuronal plasma membranes promotes the auto-activation of the classical pathway, increasing locally the availability of C1q and C3 that would, in turn, favor the reversal of GLAST/GLT1 and amplify the glutamate outflow.

Quite interestingly, we found that the anti-GluA and anti-GluN antibodies had opposite effects on the complement-induced releasing activity, which strongly depended on the impact of the antibodies on the “in-out trafficking” of the targeted receptors. Specifically, incubation of synaptosomes with commercial anti-GluA2 or anti-GluA3 antibodies (to mimic the autoantibodies) stabilized the AMPA receptors in synaptosomal plasma membranes, reducing their internalization. In these terminals, the number of presynaptic AMPARs in membranes increased, and the glutamate outflow elicited by complement from the terminals bearing the presynaptic anti-GluA-AMPAR complexes was amplified. Note that the amplification of the complement-evoked releasing activity was not observed when synaptosomes were exposed to the C1q-depleted complement, which is consistent with the involvement of the classical pathway. Furthermore, the antibody-induced amplification of the complement-mediated releasing activity was abrogated by the selective AMPA antagonist NBQX, consistent with the conclusion that the antibody-induced stabilization of AMPA receptors in plasma membranes amplified the complement-releasing activity. As a matter of fact, we propose that the anti-GluA/AMPA receptor complex could promote the auto-activation of the classical pathway, locally increasing the availability of C1q and C3, which in turn amplifies the glutamate outflow (Fig. **3**).

This experimental approach was also applied to the NMDA autoreceptors located on hippocampal glutamatergic nerve endings. Again, synaptosomes were incubated with antibodies recognizing the outer sequences of the receptor GluN subunits to assemble anti-GluN/ GluN subunit complexes in synaptosomal plasma membranes. Differently from what was observed with the AMPA receptors, however, the incubation of synaptosomes with anti-GluN1 or anti-GluN2 antibodies accelerated the internalization of the NMDARs, reducing their number in the presynaptic neuronal plasma membrane. In these particles, the complement released less efficiently glutamate with respect to the untreated synaptosomes, confirming that the complement-induced releasing activity also includes an NMDA-mediated component, as already discussed (see section 3.1) [78].

The antibody-induced activation of the complement-releasing activity was not restricted to the ionotropic glutamate receptors but was also observed when investigating the impact of antibodies recognizing the outer aminoacidic sequences of metabotropic receptors, including the CCR5 receptor that is targeted by the chemokine CCL5 (C-C motif ligand 5, Regulated upon Activation, Normal T cell Expressed and Secreted chemokine, RANTES) to mediate immune-responses [93]. CCR5 (C-C motif receptor 5) exists in human and mouse cortical glutamatergic synaptosomes and sustains the CCL5-mediated cascade of events leading to glutamate exocytosis [77]. Preincubation of synaptosomes with an antibody recognizing the NH_2_ terminus of the CCR5 protein abolished the CCL5-induced releasing activity, indicating that the antibody interferes with the CCL5/CCR5 interaction, silencing the CCL5-mediated activation of glutamate outflow. Comparable effects were obtained when synaptosomes were exposed to an antibody recognizing the COOH terminus of CCR5, suggesting that, whatever the targeted aminoacidic sequence, anti-CCR5 antibodies behaved as receptor antagonists to prevent the CCR5-mediated releasing activity. Different outcomes, however, were detected when studying the impact of the anti-CCR5/ CCR5 complex on the complement-evoked releasing activity [93, 94]. The presence of the anti-NH_2_-CCR5 antibody complex facilitated the complement-induced releasing effect in a C1q-dependent manner, consistent with the involvement of the classical pathway. On the contrary, the complement-induced releasing activity was unmodified in anti-COOH-CCR5 antibody-enriched synaptosomes, possibly, we hypothesized, because the antibody-antigen complex was inaccessible to complement. On the whole, these results confirmed that the presence of antibody-antigen complexes at the outer side of the synaptosomal plasma membrane could “*per se”* trigger the classical pathway of activation of the complement complex, leading to changes in synaptic communications and even promoting excitotoxic events. Taking into consideration that neutralizing antibodies against CCR5 are used in therapy for the management at the early stage of the acquiring human immunodeficiency virus (HIV) infection [95], our findings could unveil cellular events that support the onset of unpredictable adverse central effects in patients receiving the anti-CCR5- treatments or that, if infected with the HIV1 virus, produce circulating natural anti-CCR5 antibodies.

To conclude, the data revised in this section highlights cellular events through which antibody/antigen complexes might favour an anomalous auto-activation of complement and favour central excitotoxicity and ongoing neurological impairments. Since the use of antibodies in therapy is an emerging approach nowadays for the cure of several disorders, these observations must be taken into consideration to predict the onset of central side effects or even adverse reactions that could affect patients receiving the immunotherapies.

### 3.3 Complement and Glutamate Receptors Cross-talk: Is it Metamodulation?

The term “metamodulation” or “modulation of modulation” describes a fine mechanism of tuning neurotransmission that involves colocalized receptors/proteins that functionally interact with each other [66]. Briefly, by activating its own receptors, a neurotransmitter can exert a direct effect (*i.e*., modulation) on chemical transmission but also can indirectly tune (*i.e*., metamodulate) the activity of other, colocalized or even physically associated receptors, which are targets of another transmitter. One of the main consequences of meta-modulation is the identification of new druggable targets to tune receptors for which there is a lack of orthosteric/ allosteric ligands to be translated into clinic for therapeutic purposes.

Among the receptors susceptible to metamodulation there are the glutamate receptors (*i.e*., both the metabotropic and the ionotropic ones), which are often “metamodulated” by by-standing receptors/proteins to control their in-out trafficking in plasma-membranes as well as their responsiveness to agonists [84]. We propose that the term “metamodulation “well describes some functional interactions described in the literature linking complement and glutamatergic receptors and might provide innovative approaches for therapeutic interventions to manage ongoing glutamatergic dysfunctions. To support this view, this section briefly summarizes some results in the literature concerning the functional interactions connecting complement and glutamate receptors that, we propose, can be classified as “metamodulation.”

This is the case of kainate receptors. Complement proteins (specifically those belonging to the C1 class) were reported to modulate kainate receptors in the cerebellum and in the hippocampus by implementing the recruitment of the receptor subunit proteins in plasma membranes and by concomitantly modulating their functional outputs [1, 40]. In these studies, C1 behaved as synaptic organizers, able to regulate the formation and maintenance of synaptic connections by recruiting kainate receptors at the active synaptic contacts. Specifically, C1ql2 and C1ql3 proteins, which are actively released by presynaptic terminals, were shown to influence kainate receptors at the mossy fibre-CA3 synapses by interacting with the NH_2_-terminal domains of the GluK2 and GluK4 subunits. These interactions were proposed to subserve the correct “organization” of glutamatergic synapses but also to de-synchronize them, specifically when C1q proteins were pathologically overexpressed and could alter the cellular insertion and function of kainate receptors.

Notably, since kainate receptors also exist presynaptically and favours glutamate exocytosis [91, 96], it is conceivable to propose that complement can favour their insertion in presynaptic membranes, amplifying their releasing activity. The possibility has not been investigated so far but surely deserves attention since it could unveil a new mechanism subserving central excitotoxic events associated with the accumulation of complement components in selected brain areas [97]. The hypothesis is even more attractive when considering that data in the literature indicates that the complement-kainate receptors interaction occurs bidirectionally since, reciprocally, activation of kainate receptors controls the complement-induced neurotoxicity in oligodendrocytes [98]. The complement-kainate cross-talk could, therefore, represent a redundant system through which complement proteins and kainate receptors cooperate to sensitize cells to excitotoxicity.

Besides kainate receptors, the AMPA receptors could also be viewed as suitable partners/targets of the complement system. In this case, however, the available data suggests that the complement -AMPA receptors cross-talk relies on and requires the concomitant presence of bridging protein(s) to favour the protein/receptor interaction. We are referring to NPx, which is an accessory protein of the AMPA receptors that also colocalizes and is intimately connected to some complement proteins (specifically the C1q components, see section 3.2) [43]. By bridging C1q and the AMPA receptors, NPx can be potentially relevant to synaptic pruning, perhaps sustaining maladaptation of glutamatergic transmission. Another protein that can stabilize AMPA receptor-complement “metamodulation” is phosphatidylserine, which is a partner of annexin A and controls the interaction of SNARE proteins with membranes. Phosphatidylserine colocalizes presynaptically with C1q and the colocalization peaks during development when synaptic pruning is maximally active to eliminate weak synapses [35]. Phosphatidylserine also influences the clustering and the releasing activity of AMPA receptors [99], and taking into consideration that these receptors also locate presynaptically in cortical and hippocampal glutamatergic nerve endings, the possibility exists that phosphatidylserine might stabilize complement-AMPA receptor interaction at this side of the synaptic contact. As discussed below (see section 3.3), the complement-evoked release from cortical synaptosomes significantly increases in synaptosome-bearing anti-GluA2/GluA2 or anti-GluA3-GluA3 complexes. Whether pentraxin and even phosphatidylserine might play a role in favouring the complement-AMPA receptor interaction and the consequent amplification of the releasing activity is an attractive hypothesis that must be verified in the next future.

Differently from the AMPA/kainate receptors, the possibility that complement “meta-modulates” NMDA receptors have been so far poorly investigated. Some recent findings, however, support this view. In a recent manuscript, Bortolotto and colleagues [86] demonstrated that the mGluR5-dependent long-term depression (LTD) elicited by oligomeric β amyloid peptide in hippocampal slices depends on NMDA-mediated signalling and it is reduced by antagonizing the C5aRa1 receptor. Based on these findings, the authors proposed the existence of a signalling axis linking the mGlu5, the NMDA, and the C5a receptors, which might have a role in the cascade of events leading to synaptic alterations upstream of the overt synapse degeneration. The authors concluded that the amyloid β peptide weakens synaptic transmission (measured as altered long-term depression) *via* a mechanism involving the synergistic effects of NMDA and mGlu5 receptors that requires the concomitant activation of the C5aRs, indirectly hypothesizing a mGluR5-NMDAR-C5aR interaction. This pathway, of course, might not be specific to Alzheimer’s disease but also could underlie the cellular events that typify other neurodegenerative disorders. Another complement component that emerged as a potential candidate to “metamodulate” NMDA receptor-mediated signalling is C3. The altered expression of the GluN2B subunit in the spinal cord of rats suffering from sciatic ligation was shown to be paralleled by altered expression of the C3/C3 receptor axis, suggesting that these complement components could participate in the mechanism of pain sensitization and unveiling new druggable targets for pain managements [15, 100].

Finally, also the delta (GluD1-D2) receptors were proposed to cross-link complement. The hypothesis was first inferred by Matsuda and collaborators [38, 40] that proposed the existence of a trans-synaptic tripartite complex consisting of one unit of the Cbln1 hexamer (a complement component of the C1 class), the monomeric NPx, and the GluD2 dimers. The author hypothesized that the trimer was distributed trans-synaptically, bridging the pre to the postsynaptic side of the synaptic contact.

Besides the ionotropic glutamate receptors, the metabotropic ones must also be taken into consideration for possible mechanisms of “metamodulation” involving complement. In this view, besides the evidence suggesting the mGluR5-NMDAR-C5aR that participates in the loss of memory and synaptic weakening that typify dementia [86], Spurrier and colleagues in 2022 demonstrated that treatment with the mGlu5 silent allosteric modulator BMS-984923 reduces the β amyloid-induced changes of synaptic signalling also preserving the glutamate transmission to physiological level [87]. The novelty of the study was that the treatment also reduced the synaptic accumulation of C1q at both PSD95-positive postsynaptic compartments and synapsin 2 immuno-positive presynaptic side in the hippocampal dentate gyrus, consistent with a reduced synaptic tagging. In other words, modulating the mGlu5 receptors directly affects the NMDAR-mediated effects but also indirectly controls the accumulation and the pathological synaptic pruning elicited by C1q, which is fundamental to the progression of the disease.

Attention also focussed on the mGlu1 receptor (mGlu1R), the activation of which facilitates the local translational machinery of the C1q mRNA, eliciting an increased production of the C1q protein, even in synaptosomal preparations, amplifying synaptic pruning [101]. The strict correlation linking the mGlu1 activation and the C1q production was supported by the finding that the *in vivo* administration of a specific mGlu1 inhibitor, the compound JNJ16259685, attenuated the accumulation of C1q in the hippocampal synaptosomal fractions. The functional correlation linking activation of mGlu1Rs and the over production of C1q was confirmed using mGluR1 knock-out mice. These animals did not show C1q upregulation as before [90]. Interestingly, the mGlu1R -C1q interaction was proposed to localize at glutamatergic synapses and to have a main role in their elimination by amyloid fibrils. More recently, these authors demonstrated the main role of the type 2 protein phosphatase in the mGlu1-C1q interaction [102].

Lastly, complement might “metamodulate” glutamate receptors by interacting with anchoring proteins that favor the insertion and stabilize AMPA and NMDA receptors in plasma membranes, potentially affecting their participation in synaptic communication. These events are expected to occur preferentially in the post-synaptic density and would deserve attention since they might be pathogenic.

To conclude, complement constituents can either stabilize receptor proteins in membranes but also behave as natural linkers, bridging these receptors to other entities and influencing the functional receptor outcomes.

## 4 THERAPEUTIC STRATEGIES TO TARGET THE COMPLEMENT SYSTEM

The literature revised so far suggests that dysregulation, inadvertent activation, or overexpression of complement components contribute in different ways to impair glutamatergic transmission, being pivotal or subserving the onset and the course of neuropsychiatric and neurodegenerative disorders. Accordingly, to this view, complement-mediated dysregulation of glutamate transmission was recently called upon the development of chronic (*i.e*., Alzheimer’s disease, amyotrophic lateral sclerosis, Huntington’s disease, Parkinson’s diseases) or acute (*i.e*., traumatic brain and spinal cord injury) neurodegenerative diseases, neuropsychiatric pathologies (*i.e*. schizophrenia and mood disorders) and neuroimmune and inflammatory disorders (*i.e*. Multiple sclerosis, neuromyelitis optica, autoimmune encephalopathies) [8, 9, 12, 14, 29].

These findings increased the interest of pharmacologists on the possible application in therapy of compounds that, by controlling the mechanisms of complement auto-initiation or by modulating/mimicking endogenous complement modulators, could normalize the impact of complement on central glutamatergic innervation. Some of the identified molecules are in preclinical studies, and the available data unveiled promising results (*i.e*., DF3966A, a C5a antagonist [103, 104] that would support their entry into clinical trials. For instance, DF3966A was recently shown to protect patients from chemotherapy-induced peripheral neuropathy [105]. Taking into consideration the role of hyper-glutamatergic in spinal pain perception, the possibility that this ligand might recover the complement-induced modulation of glutamate to physiological levels in this region is attractive and would deserve investigation.

Other complement modulators have already successfully passed the preclinical studies and are now under evaluation in ongoing clinical trials (at different phases) for their application in the clinic for the management of specific central disorders, and few of them have successfully passed the phase three and are approved for the management of specific disorders. Besides the administration of high doses of immunoglobulins, the therapeutics already in the clinic include the humanized antibody ANX005, which is raised against the C1q component; pegcetacoplan, a C3 inhibitor of the compstatin family; antibodies such as Eculizumab, Ravulizumab, Tesidolumab which target C5, zilucoplan, which prevents the C5 dissociation. Despite the promises of new therapeutic applications for ameliorating central pathologies ranging from inflammation autoimmune diseases to neuropsychiatric and neurodegenerative disorders, their use in therapy, however, must be regarded with attention because of the possible onset of adverse-side effects that are often observed after prolonged use in pharmacological regimens. These adverse drug reactions are mainly due to the interference of certain complement modulators with mechanisms of host defence against infections, which might, in turn, induce unwanted susceptibility to infections. In the last decade several reviews have been dedicated to the use of complement ligands in therapy and to their unwanted side effects [15, 103, 104, 106] and can be consulted for more information.

## CONCLUSION

In the last two decades, several lines of evidence indicated the presynaptic distribution of the complement components C1q, C3, and C5 in selected regions of the CNS and proposed their involvement in events subserving synaptic refinement in physiological and pathological conditions. The progress in understanding the central role of complement in the CNS, however, increased in recent years, and it is nowadays recognized that complement (specifically the C1q and the C3 components) also exerts presynaptic “non-canonical” effects that, based on the recent literature, specifically modulate synaptic transmission. Convinced that these events could have a prominent role in determining the dynamic processes that define the efficiency of synaptic communication in both physiological and pathological conditions, this review is dedicated to revising the available literature that proves the ability of complement and specifically of its components C1q, C3 and C5a to modulate presynaptically glutamate transmission.

The observations described here are consistent with the conclusion that complement releases specifically glutamate from nerve endings but also “metamodulate” glutamate receptors (having both presynaptic and postsynaptic localization), favouring the coupling of colocalized receptors/ misfolded proteins and even stabilizing their insertion in the neuronal plasma membrane. We have also revised data unveiling mechanism(s) of complement autoactivation that could further amplify the complement-induced release of glutamate, being detrimental in the course of autoimmune diseases typified by the production of autoantibodies or even accounting for adverse reactions during antibody therapy.

In our opinion, these findings implement our knowledge of the impact of complement in the CNS, posing the basis for understanding the molecular and cellular events which participate to the progression of those central neurological diseases for which complement is a key central contributing factor. Deepening the knowledge of the “non-canonical” role of complement in dictating the efficiency of excitatory transmission would suggest new innovative therapeutic interventions for the management of central diseases that lack so far efficacious therapeutic approaches.

## LIST OF ABBREVIATIONS

CCL5: C-C Motif Ligand 5
CCR5: C-C Motif Receptor 5
CNS: Central Nervous System
dl-tBOA: DL-threo-beta-Benzyloxyaspartate
EAAT: Excitatory Aminoacid Transporter
EPSP: Excitatory Postsynaptic Potential
FACS: Fluorescence Activated Cell Sorting
GLAST: Glutamate Aspartate Transporter
GLT-1: Glutamate Transporter-1
HIV: Human Immunodeficiency Virus
ko: Knock-out
LTD: Long-term Depression
MAC: Membrane Attack Complex
mGluR1: Metabotropic Glutamate Receptor Type 1
mGluR5: Metabotropic Glutamate Receptor Type 5
NPx: Neuronal Pentraxin
Nx: Neurexin
PSD95: Post-synaptic Density Protein 95
RGC: Retinal Ganglion Cells
SEPT: Septin
SLC1: Solute Class 1 of Transporters
SNAP25: Synaptosomal-associated Protein, 25kDa
Stx-3: Syntaxin-3
SV2: Synaptic Vesicle Glycoprotein 2
SYN: Synaptophysin
VAMP2: Vesicle-associated Membrane Protein 2
VGluT: Vesicular Glutamate Transporter
wt: Wild Type
